# Fixation of Small Osteochondral Fragments in a Comminuted Distal Humerus Fracture with Magnesium Bioabsorbable Screws: A Case Report

**DOI:** 10.7759/cureus.3752

**Published:** 2018-12-19

**Authors:** Cemil Aktan, Mehmet B Ertan, Adil Turan, Ozkan Kose

**Affiliations:** 1 Department of Orthopaedics and Traumatology, University of Health Sciences, Antalya Education and Research Hospital, Antalya, TUR

**Keywords:** humerus fracture, bioabsorbable screw, internal fixation

## Abstract

The treatment of comminuted distal humeral fractures with free osteochondral fragments is challenging. Osteochondral fragments should be retained whenever possible and secured with implants buried beneath the articular surface to obtain a uniform articular surface. Headless compression screws and K wires are commonly used for this purpose. However, certain complications have been reported with these fixation implants in case of the non-union and osteolysis of the fragments such as migration and cartilage damage. Fixation of osteochondral fractures in distal humeral fractures using bioabsorbable implants has been rarely reported in the current literature. Herein, a patient who sustained a comminuted distal humeral fracture with multi-fragmentary osteochondral fragments is presented, and treatment with magnesium bioabsorbable compression screws is discussed.

## Introduction

Distal humeral fractures are relatively rare injuries that constitute approximately 2% of all fractures [1]. Most of these fractures occur due to high energy trauma in young males. Because they involve the articular surface and usually cause an instable elbow, surgical treatment is necessary for the recovery of elbow functions in the majority of cases. Anatomic reduction of the joint surface and stable fixation of the distal humeral columns, which allow early movement and rehabilitation, are widely accepted treatment principles in distal humeral fractures. The most commonly used methods are stabilization with medial and lateral colon plates and screws [2].

However, in some cases, these fractures are highly comminuted, extending across the whole distal articular surface and producing small osteochondral fragments with various amount of impaction. In case of such articular surface comminution, the treatment is more challenging for surgeons. Osteochondral fragments should be retained whenever possible and secured with implants buried beneath the articular surface, such as countersunk mini-fragment screws, headless variable-pitch screws, or small threaded K-wires [3].

Despite proper fixation, small osteochondral fragments may not heal and osteolysis may happen due to complete loss of their blood supply [4]. Thereafter, emerging metal implants embedded in the bone cause damage to the facing healthy cartilage, which may induce the development of osteoarthritis. Migration of the K wires can also be seen. Moreover, the removal of these implants during any revision surgery poses another problem. Fixation of these osteochondral fragments with bioabsorbable screws may contribute to the solution of all the above-mentioned problems.

In the current literature, there is a limited number of cases reporting the fixation of intraarticular fragments with bioabsorbable screws. Herein, a patient who sustained comminuted distal humeral fracture with multi-fragmentary osteochondral fragments is presented, and treatment with magnesium bioabsorbable compression screws is discussed.

## Case presentation

**Figure 1:**
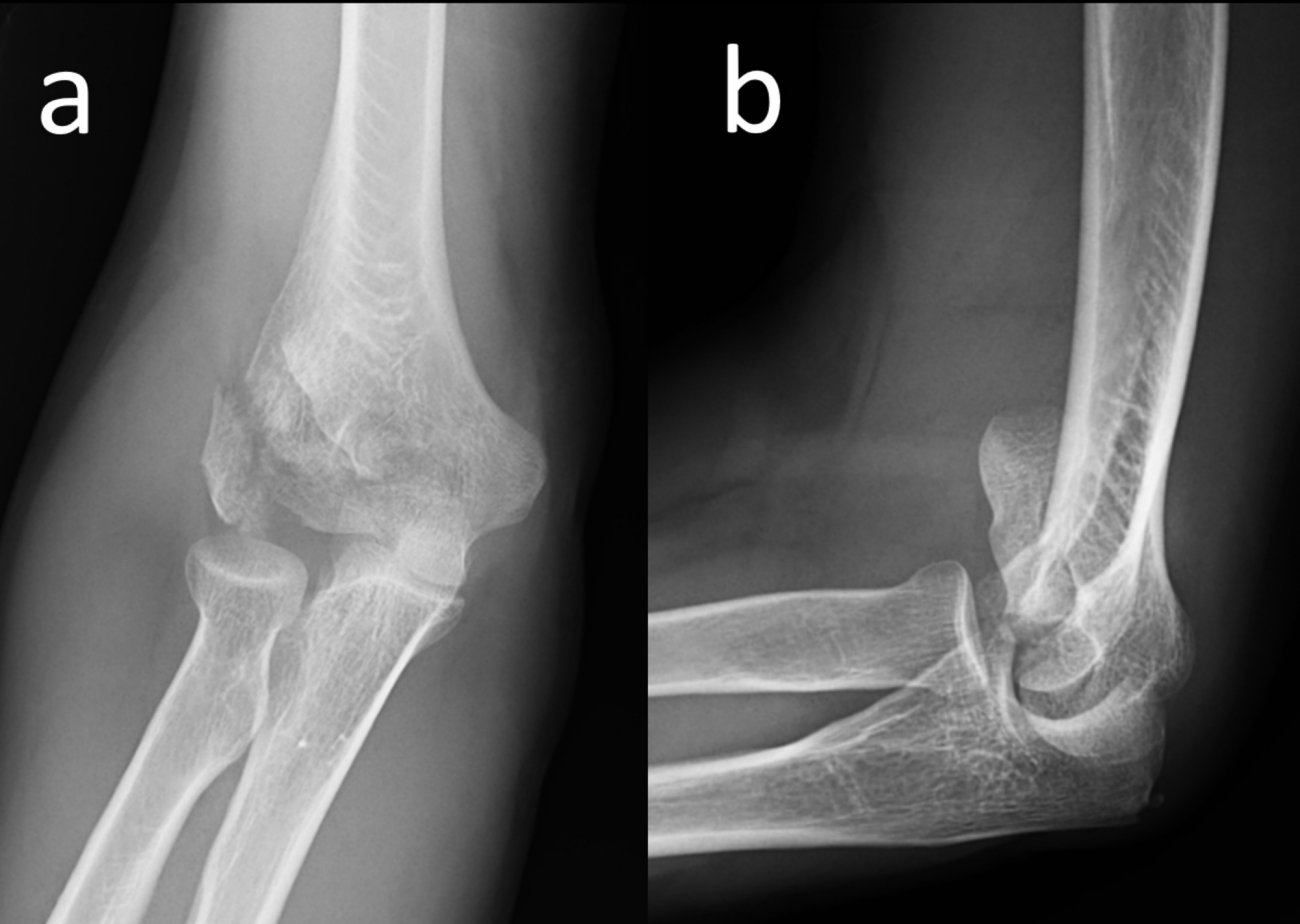
Preoperative elbow radiograph Anteroposterior (a) and lateral (b) elbow radiographs of the patient at initial admission, showing comminuted distal humeral fractures with articular involvement.

We report a 50-year-old male patient who was admitted to our emergency department after falling from a height onto his elbow. On physical examination, his elbow was swollen and tender. The active and passive elbow range of motion was painful and limited. The neurovascular examination of the upper extremity was normal. Direct radiographic examination of the elbow showed a distal humeral fracture with comminution of the capitellum and lateral column (Figure 1).

**Figure 2:**
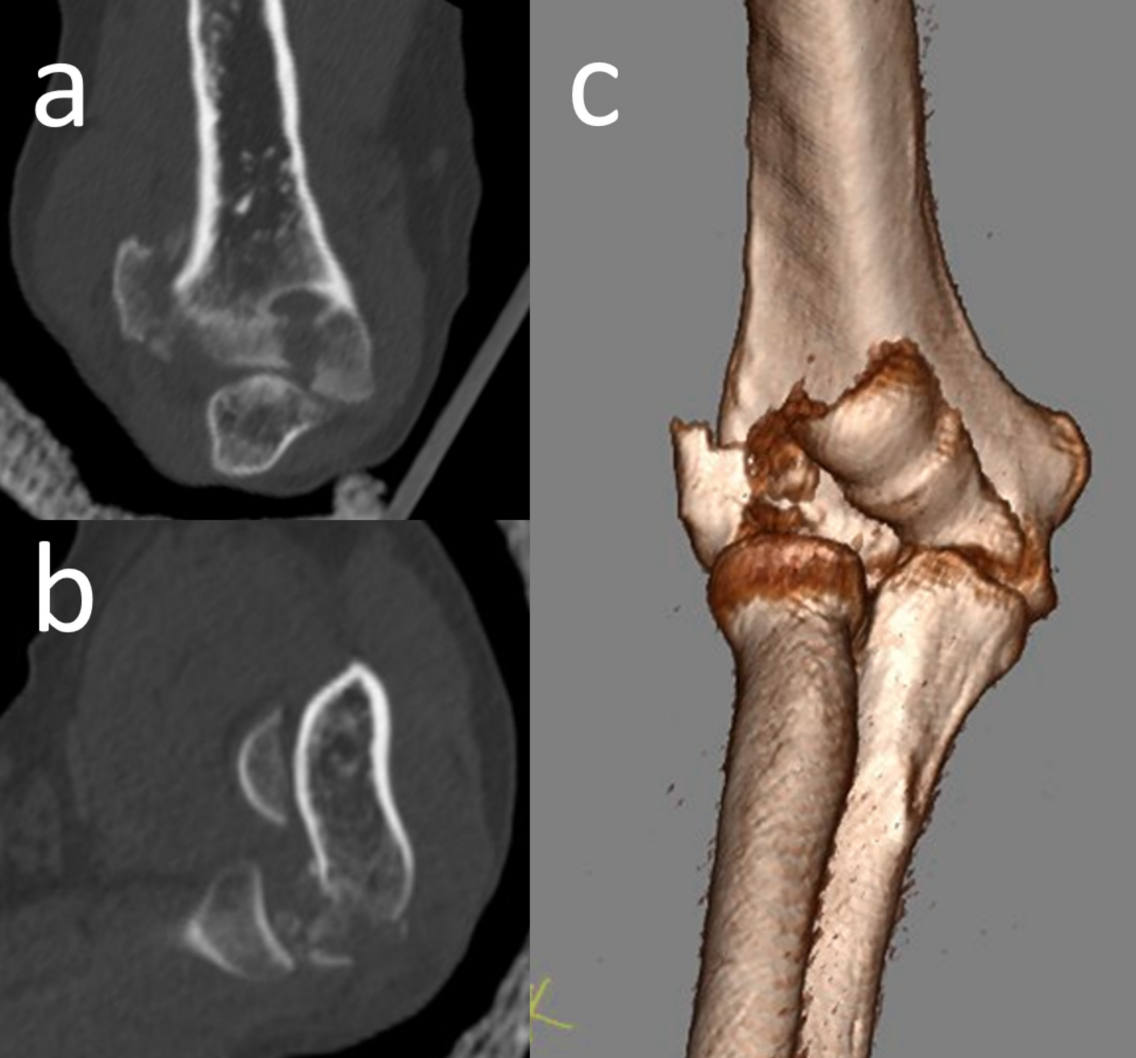
Computerized tomography of the elbow Computerized tomography of the elbow. Coronal (a) and sagittal (b) sections demonstrated the free osteochondral fragments. 3D CT appearance (c) of the fracture. 3D: three-dimensional; CT: computed tomography

In order to understand the extent of the comminution, computerized tomography (CT) imaging was performed. The CT examination showed fragmentation of the capitellum and a fracture extending to the lateral column (Figure 2).

**Figure 3:**
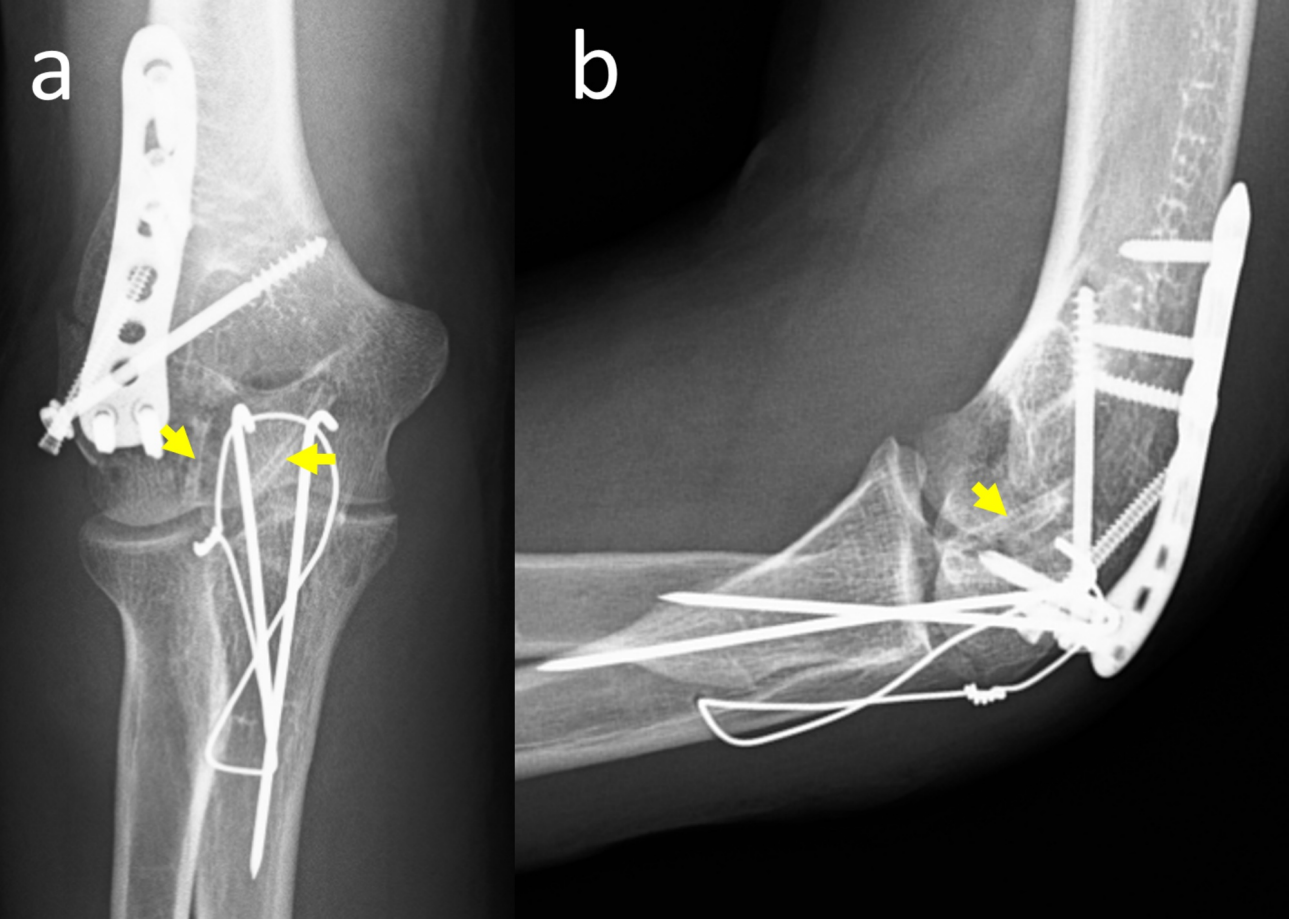
Postoperative radiographs Anteroposterior (a) and lateral (b) elbow radiographs immediately after operation. Yellow arrows show the magnesium bioabsorbable screws.

Surgical fixation of the fracture was planned based on imaging findings. Under general anesthesia and tourniquet control, a posterior surgical approach with olecranon osteotomy was used for the exposure of the fracture. The articular surface was reduced and fixed with two 2.7 mm diameter magnesium bioabsorbable screws (MAGNEZIX® CS, Syntellix AG, Hanover, Germany). The lateral column was fixed with an anatomic lateral column plate. It has been paid attention that the two materials (titanium and magnesium) did not touch each other physically. The olecranon osteotomy was fixed with the tension band wiring technique (Figure 3).

**Figure 4:**
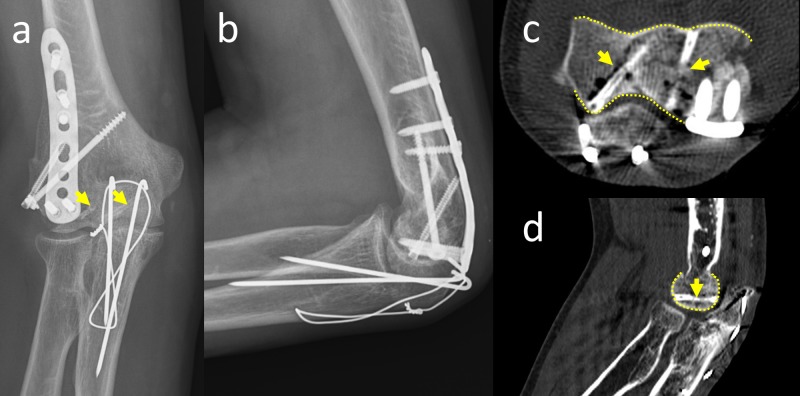
Final follow-up radiographs and CT Anteroposterior (a) and lateral (b) elbow radiographs at the fourth-month follow-up. Axial (c) and sagittal (d) CT examination demonstrated the union of fracture and perfect articular surface (yellow dotted lines show the smooth articular surface and yellow arrows show the screws). CT: computed tomography

After the operation, the patient was immobilized in an above-elbow plaster cast for 10 days to facilitate the subsidence of edema around the elbow. Thereafter, the plaster cast was removed and the active elbow range of motion exercises was initiated. At the first month follow-up, the elbow range of motion was between 20 and 110 degrees. Supination and pronation of the forearm were in the normal range. At the fourth-month follow-up, the fracture was united and the elbow range of motion was nearly normal, between 5 degrees and 130 degrees (Figure 4). The Mayo elbow performance score was 100.

## Discussion

The treatment of comminuted distal intra-articular humeral fractures is difficult, particularly in the presence of osteochondral fragments comprising the articular surface. It is extremely important to properly fix these fragments to obtain a uniform articular surface. If the osteochondral fragment is too small, which is not amenable for fixation, excision can be performed, but it is recommended to retain all fragments containing a significant part of the joint. Screws of either the lateral or the medial column plate often fix these fragments inadequately and sometimes cannot provide any fixation. Therefore, these fragments should be fixed with independent implants. The most commonly used fixation techniques are the use of headless compression screws if the fragment is large enough or the thin threaded K wires if the fragment is very small [5].

In the presented case, magnesium bioabsorbable screws (Alloy: MgYREZr) were used for the fixation of osteochondral fragments. This alloy contains magnesium, zirconium, yttrium, and rare earth elements. Magnesium alloys are relatively new bioabsorbable biomaterials used in orthopedic surgery. Previously, several preclinic and experimental studies showed adequate degradation characteristics and mechanical integrity of these biomaterials for fracture fixation. Rare earth element-based alloys, such as WZ21, were reported to be suitable even for growth plate applications, with their decreased gas formation and prolonged maintenance of mechanical stability without disturbing the growth plate [6]. Thereafter, screws and pins made up of the MgYREZr alloy were manufactured and became clinically available on the market. These screws were first used in metatarsal osteotomies in hallux valgus deformity corrections [7]. Then, the indications were broadened and successful results were also reported in various fracture types and indications. Kose et al. reported good functional results with a complete union in medial malleolar fractures [8]. In vivo and in vitro experimental studies have shown that the degradation products of magnesium screws do not harm the surrounding cartilage. Recently, Gigante et al. performed fixation of anterior cruciate ligament avulsion fractures with magnesium screws and reported good results without any surrounding articular cartilage deterioration [9]. Biber et al. described a patient with an osteochondral fracture of the humeral capitellum treated with a bioabsorbable magnesium screw, which resulted in fracture union without any complications [10]. All these previous successful results suggest that magnesium bioabsorbable screws can be used safely in intraarticular fracture fixation.

In the fourth month, no gas formation was observed around the screws on direct radiographs of the elbow in our patient. However, the CT examination showed a slight accumulation of gas around the screws, which was clinically insignificant. The quality of reduction of the joint surface was adequate and smooth without the protrusion of the screws. Union of the osteochondral fragments and degradation of the screws could be seen on axial and sagittal CT views.

## Conclusions

Because these screws are bioabsorbable and replaced by native bone, they eliminate some of the obstacles caused by metal screws for joint surface fixation. First, in case of osteolysis of the fragment, there will be no protruding metallic screw head to damage the facing healthy cartilage. Second, it provides a convenient environment at the time of any secondary revision surgery, as it turns into normal bone. Third, it reduces the extensile surgical approach for the removal of intraarticular screws. The use of magnesium bioabsorbable screws is an advantageous option for distal humeral intraarticular osteochondral fragment fixation.
